# *Phrynahamzaoglui* Koç & Budak (Caryophyllaceae), a new species from Central Anatolia, Turkey

**DOI:** 10.3897/phytokeys.109.24609

**Published:** 2018-09-19

**Authors:** Murat Koç, Ümit Budak

**Affiliations:** 1 Boğazlıyan Vocational School, Bozok University, 66900, Yozgat, Turkey Bozok University Yozgat Turkey; 2 Department of Biology, Faculty of Science and Arts, Bozok University, 66200, Yozgat, Turkey Bozok University Yozgat Turkey

**Keywords:** *
Phryna
*, Malatya, taxonomy, new species

## Abstract

A new species *Phrynahamzaoglui* was discovered in Hekimhan (Turkey, Malatya province) where it grows on hillsides. The *P.hamzaoglui* and *P.ortegioides* were compared with each other in terms of their general morphology and seed micromorphology. Description, distribution, illustration and conservation status of the new species are given. Seed lateral and surface, cells, anticlinal cell walls, periclinal cell walls structures were examined by scanning electron microscopy. The images were captured with the 500×, 100×, and 40× objective lens for the details.

## Introduction

The family Caryophyllaceae Juss. includes approximately 88 genera and 3000 species that are almost entirely distributed throughout the northern hemisphere and occasionally throughout the southern hemisphere. The main distribution area of this family is the mediterranean phytogeographical region (Lawrence 1951, Rabeler and Hartman 2005). This family is split into three subfamilies, namely Alsinoideae (DC.) Fenzl, Caryophylloideae and Paronychioideae (A.L.Juss.) Meisn (Bittrich 1993). The Caryophylloideae subfamily is divided into the tribes *Caryophylleae, Drypideae* Fenzl and *Sileneae* DC. (Bittrich 1993).

The genus *Phryna* (Boiss.) Pax & K.Hoffm. belongs to the tribe Caryophylleae that exist in subfamily Caryophylloideae of the family Caryophyllaceae. A specimen of this genus collected from Turkey was last revised by Huber-Morath in his work entitled “Flora of Turkey and the Aegean Islands” which was published on 1967. In this revision, the existence of a species belonging to this genus was reported (Huber-Morath 1967), the genus being similar to *Ankyropetalum* Fenzl, *Acanthopyllum* C.A.Mey., *Allochrusa* Bunge, *Gypsophila* L. and *Bolanthus* (Ser.) Rchb. (Huber-Morath 1967, Barkoudah 1962).

## Materials and methods

Some interesting specimens belonging to the genus *Phryna*, in which their main distribution area is the Irano–Turanian phytogeographical region, were collected from Malatya province. As a result of examining the literature (Barkoudah 1962, Huber-Morath 1967) and herbarium specimens, it was found that the collected specimens resemble *Phrynaortegioides* but differ from this species in terms of calyx length, calyx teeth length, petal colour, petal calyx ratio and capsule calyx ratio. Flower, petal and capsule pictures were taken with an OLYMPUS SZX-16 Stereomicroscope, DP 72 digital camera and seed surface images were taken by a Quanta Feg 450 scanning electron microscope (SEM) at Bozok University Research and Application Centre. The vegetative parts were measured with a ruler with 0.5 mm accuracy and the floral characteristics were studied using an ocular micrometer.

## Taxonomic treatment

### Phryna hamzaoglui

Taxon classificationPlantaeCaryophyllalesCaryophyllaceae

Koç & Budak sp. nov.

urn:lsid:ipni.org:names:60477028-2

Figs 1 , 2 , 3

#### Diagnosis.

*Phrynahamzaoglui* is related to *P.ortegioides*. It differs from *P.ortegioides* mainly because it has a calyx 4–5.5 mm long, teeth 2–2.5 mm long (not 3–4 mm long, teeth 1–2 mm long); petals completely white not purple veins, as long as calyx (not white with purple veins, 1.5–2 times calyx); capsule including calyx (not longer than calyx); seeds ventral and dorsal cells margine S-undulate (not V-Undulate) (Table 1).

**Table 1.** Diagnostic characters between *Phrynahamzaoglui* and *P.ortegioides*.

	* P. hamzaoglui *	* P. ortegioides *
Calyx	4–5.5 mm long, teeth 2–2.5 mm long	3–4 mm long, teeth 1–2 mm long
Petal	Completely white not purple veins, as long as calyx	White with purple veins, 1.5–2 times calyx
Capsule	Including calyx	Longer than calyx
Seeds	Ventral and dorsal cells margine S-undulate	Ventral and dorsal cells margine V-undulate

#### Type.

TURKEY. B6 Malatya: Between Hekimhan and Hasançelebi town, 3 km from Hekimhan, 1100 m a.s.l., hillside, 10 October 2015, Koç 2353, Hamzaoğlu & Budak (holotype ANK; isotypes ANK, GAZI, Bozok Univ. Biology Dept. Herbarium).

#### Description.

Perennial herb. Stems woody caudex, dichotomously forked stems, 30–35 cm, base 1–1.2 mm diam., entirely mixed eglandular and with glandular hairs; internodes 13–20 mm, with glandular hairy. Leaves linear, 6–15 × 0.9–1.1 mm, with glandular hairs, 3–nerved, greenish, solid; acute at apex; sheath membranous, 0.2–0.3 mm long, having glandular hairy. Inflorescence mostly solitary, forming lax terminal racemes. Flowers sessile or subsessile. Bracts lanceolate, 3–4 × 0.8–1 mm; acute at apex. Bracteoles 2–3 pairs; acute at apex. Calyx narrowly campanulate, having densely glandular hairy, 4–5.5 × 1–1.2 mm, with 5 projecting ribs; teeth lanceolate, 2–2.5 mm long; acuminate at apex. Petals oblanceolate, 4–6 × 0.7–0.9 mm, as long as calyx, entirely white not purple veins; rounded at apex; base cuneate. Stamens 4; filaments 3–4 mm long. Styles 2, 3–4 mm long. Capsule oblong, 3.5–5 × 1–1.2 mm, 2–seeded, including calyx.

**Figure 1.**
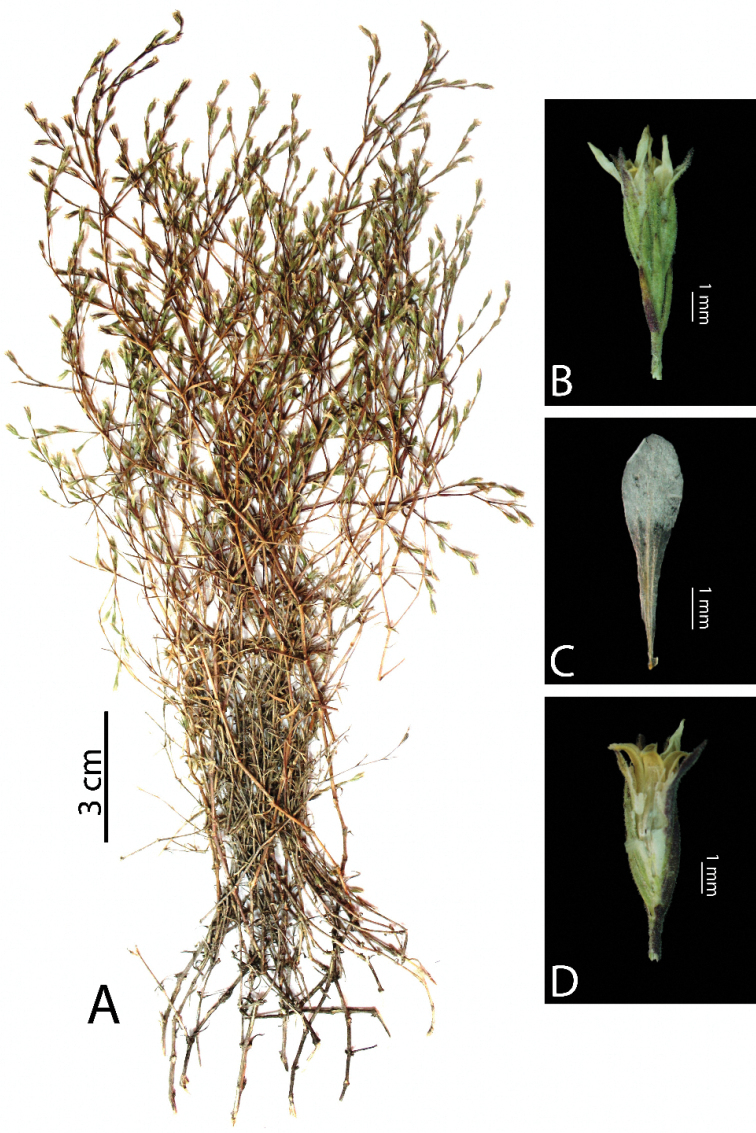
*Phrynahamzaoglui*. **A** habit **B** flower **C** petal **D** capsule.

#### Seed micromorphology.

**Figure 2.**
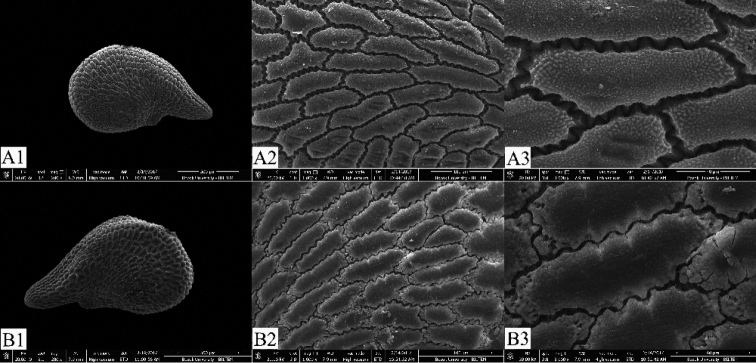
SEM photographs of the seed coat. **A***Phrynaortegioides***B***P.hamzaoglui*. Scale bars: 500 μm (**A1, B1**); 100 μm (**A2, B2**); 40 μm (**A3, B3**).

Seeds of *Phrynahamzaoglui* are comma-shaped with prominent radicle, 1–1.4 × 0.8–1 mm; blackish; lateral and dorsal surfaces tuberculate; cells subrectangle, regular; anticlinal cell walls certainly S-undulate; periclinal cell walls convex, wrinkled, upper smooth (Figure 2).

#### Etymology.

The species is named in honour of the eminent Turkish botanist Prof. Dr. Ergin Hamzaoğlu (Gazi University, Ankara).

#### Distribution and habitat.

*Phrynahamzaoglui* is an endemic species known only from the type gathering in Central Anatolia (Hekimhan, Malatya) and is an Irano–Turanian element. *Phrynahamzaoglui* prefers hillsides at an altitude of 1100 m in Malatya province.

**Figure 3.**
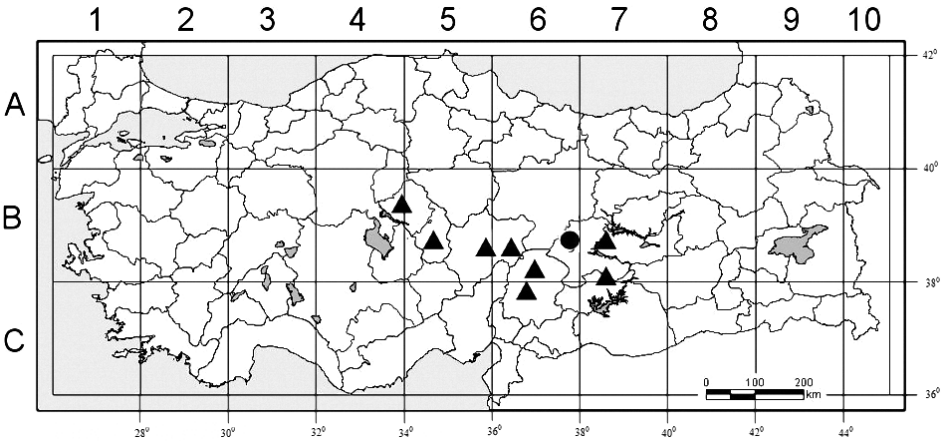
Distribution map of *Phrynaortegioides* (▲) and *P.hamzaoglui* (●) in Turkey.

#### Phenology.

Flowering from August to September; fruiting from October.

#### Conservation status.

According to current data, *Phrynahamzaoglui* grows in an area of approximately 1000 km^2^ covering the Hekimhan districts of Malatya province. The species, which has a discontinuous distribution, has been reported in only one locality in Malatya. Due to overgrazing, the habitat of this species is under threat and destruction of the species is leading to the reduction in the number of plants. For this reason, it is proposed that the species should be classified as Endangered [EN (B1a)] according to the International Union for Conservation of Nature (IUCN 2014).

#### Other specimens examined.

*Phrynaortegioides.* TURKEY. Kayseri: Kıranadardı waterfall, 1710 m, 20 July 2013, Hamzaoğlu 6889 & Koç (Bozok Univ. Biology Dept. Herbarium); Kayseri: Sarız, Büyüksöbeçimen town, 15 July 2008, Hamzaoğlu 5229 (Bozok Univ. Biology Dept. Herbarium); Adıyaman: Nemrut mountain, 2000–2200 m, 18 June 1997, Vural & Adıgüzel (GAZI, No: 8222); Elazığ: Keban Altınayva town, 1300 m, 11 August 1995, Ali.A.Dönmez 4899 & Aslantaş (GAZI); Kahramanmaraş: Elbistan, Şardağı, 1600 m, 21 August 1989, Aytaç (GAZI, No: 3012); Kayseri: Erciyes mountain, 2250–2350 m, 21 September 1993, Vural 7028, Duman, Adıgüzel & Erik (GAZI); Nevşehir: Zelve, Akdağ, 1250 m, 08 August 1989, Vural & Eyüboğlu (GAZI, No: 5487); Kırşehir: Kaman, 1200 m, 05 September 1995, Aytaç 7329 & Adıgüzel (GAZI); Kahramanmaraş: Göksu, Çardak, Binboğa mountain, Tülüce hill, 1450–1850 m, 08 August 1988, Aytaç (GAZI, No: 2537).

## Results

The *Phryna* is an endemic genus for Turkey. This genus is similar to *Petrorhagia*, *Ankyropetalum*, *Acanthopyllum*, *Allochrusa*, *Gypsophila* and *Bolanthus*. All of these genera have calyx with membranous hyaline intervals between the nerves (Barkoudah 1962, Huber-Morath 1967). *Petrorhagia* can separate from the above genera by seeds with the presence of peltate with facial hilum. While *Ankyropetalum*, *Acanthopyllum*, *Allochrusa* and *Gypsophila* have reniform seeds, *Bolanthus* and *Phryna* genera have comma seeds. In addition to these characters, the genus Phryna is distinguished from all the above-mentioned genera by having 1-4 pairs of bracteoles on the base of calyx (Barkoudah 1962).

While *Phrynaortegioides* species has a wide distribution in Turkey, the distribution of P. *hamzaoglui* is known only around Hekimhan (Malatya). The specimens of Hekimhan (Malatya) show similarity to *Phrynaortegioides* due to their having stems covered with glandular-puberulent hairy, woody caudex and flowers sessile, axillary and terminal, mostly solitary, seeds comma-shaped with prominent radicle, blackish; lateral and dorsal surfaces tuberculate; cells subrectangle, regular; periclinal cell walls convex, wrinkled, upper smooth. However, they differ from this taxon in terms of having calyx length of 4–5.5 mm, petals entirely white not purple veins and as long as calyx, capsule including calyx, seeds ventral and dorsal cells margine S-undulate (Table 1). As a result, all the obtained morphological and micromorphological data showed that the species distributed in Malatya province are new to science. The genus is still endemic for Turkey and but will be regarded as a polytipic genus.

### Key to the species of Genus *Phryna* in Turkey

**Table:** 

1	Calyx 4–5.5 mm long, teeth 2–2.5 mm long; petals completely white not purple veins, as long as calyx; capsule including calyx	*** P. hamzaoglui ***
–	Calyx 3–4 mm long, teeth 1–2 mm long; petals white with purple veins, 1.5–2 times calyx; capsule longer than calyx	*** P. ortegioides ***

## Supplementary Material

XML Treatment for Phryna hamzaoglui
